# FROM EXPANDED NEONATAL SCREENING TO THE POST-GENOMIC ERA

**DOI:** 10.1590/1984-0462/;2017;35;3;00017

**Published:** 2017

**Authors:** José Simon Camelo

**Affiliations:** a Faculdade de Medicina de Ribeirão Preto da Universidade de São Paulo (USP) - Ribeirão Preto, SP, Brasil.

After the epidemiological transition, which significantly reduced childhood mortality due to basic causes, the subjacent problem of rare diseases, and, particularly, inborn errors of metabolism emerged. The study by Romão et al.,^1^ published in this issue of *Revista Paulista de Pediatria* in 2017 about the initial clinical presentation of patients with hereditary metabolic diseases, has become important once again since it presents an updated scenario of the problem, including all of the difficulties related to the diagnosis of such conditions.

The mean age of diagnosis is around 4.3 years old. At this age for some children, irreversible consequences have already occurred, even though they have had existing symptoms since before the age of one. Diseases caused by impairment in the intermediary metabolism of small molecules usually become present earlier and have more exuberant symptomatology, which should lead to a quick diagnosis and treatment in order to prevent damage and to improve children’s quality of life (diet treatments, liver transplants, and the replacement of cofactors, among others). A study conducted at Boston Children’s Hospital shows the positive impact of neonatal screening: only 2% of the diseases detected by the expanded screening present severe outcomes, in comparison to 42% of the children diagnosed clinically. The mean intelligence quotient score (IQ) was 103±17 in the cases screened in the neonatal period, against 77±24 in cases of late diagnosis, with clear evidence of having prevented mental impairment.^2^

Another important group is late onset lysosomal storage diseases that are associated with more specific symptomatology, including dysmorphia, dysostosis, and progressive neurological changes. This group has had one of the greatest recent advances in the field, which is enzymatic replacement therapy (ERT) with products developed by biotechnology, or the use of drugs to reduce the production and accumulation of substrates.

Expanded neonatal screening, therefore, becomes a powerful tool for pre-symptomatic diagnosis. A seminal article by Watson et al., in 2006,^3^ brought a major contribution from the American Academy of Pediatrics Newborn Screening Task Force, which is associated with the American College of Medical Genetics and Genomics. Together they defined the minimum central panel of metabolic diseases to be screened in 29 diseases, chosen according to the criteria from Wilson & Jungner, who highlight the possibility of treatment, the importance for public health, and cost-effectiveness. More recently, the minimum panel added severe combined immunodeficiency and critical cyanotic congenital heart disease, giving the central panel 31 conditions.^4^ This is a relevant starting point, but it is also important that each country is aware of its own reality, especially the frequency of inborn errors of metabolism, and thus defines its own neonatal screening panel, using a responsible approach focused on damage reduction, improved quality of life for patients and families, and cost-effectiveness.^5^

More recently, platforms to diagnose lysosomal storage diseases have been made available, even though they involve unresolved ethical issues, such as the early diagnosis of diseases that could end up being mild or that could manifest later in the patients’ lives. Nevertheless it is important to define the incidence of such diseases in specific populations.^6^

This route is irreversible. Neonatal screening expansion is characterized by two great landmarks in over 50 years of history: the first one, tandem mass pre-spectrometry, when the classic screenings for phenylketonuria, congenital hypothyroidism, galactosemia, congenital adrenal hyperplasia, hemoglobinopathies, cystic fibrosis, homocystinuria, and biotinidase deficiency appeared; the second one, with technology based on tandem mass spectrometry able to diagnose many conditions simultaneously, such as aminoacidopathies, organic acidemia, and fatty acid beta-oxidation disorders, a major increase in data instructing the diagnosis, prevention and the proper handling of these conditions occurred. The difficulties of the latter are inherent and frequently require the final diagnosis to be conducted by molecular analysis. Serious considerations have been made in order to adopt the next generation of complete gene sequencing as the third landmark in neonatal screening, and such a discussion will evolve over time. However, important contributions have been made with the recent diagnosis of many hereditary conditions that were previous unknown, as well as the identification of genetic variants indicating risk for many other diseases in the same individual, and, by extension, in other family members.^7^

## References

[1] Romão A, Simon PEA, Góes JEC, Pinto LLC, Giugliani R, de Luca GR (2017). Apresentação clínica inicial dos casos de erros inatos do metabolismo de um hospital pediátrico de referência: ainda um desafio diagnóstico. Rev Paul Pediatr.

[2] Landau YE, Waisbren SE, Cha LMA, Levy HL (2017). Long-term outcome of expanded newborn screening at Boston children's hospital: benefits and challenges in defining true disease. J Inherit Metab Dis.

[3] American College of Medical Genetics Newborn Screening Expert Group (2006). Newborn screening: toward a uniform screening panel and system-executive summary. Pediatrics.

[4] Sparks SE (2013). Update on newborn screening. North Carol Med J.

[5] Jansen ME, Lister KJ, van Kranen HJ, Cornel MC (2017). Policy making in newborn screening needs a structured and transparent approach. Front Public Health.

[6] Matern D, Gavrilov D, Oglesbee D, Raymond K, Rinaldo P, Tortorelli S (2015). Newborn screening for lysosomal storage disorders. Semin Perinatol.

[7] Landau YE, Lichter-Konecki U, Levy HL (2014). Genomics in newborn screening. J Pediatr.

